# AutoPM3: enhancing variant interpretation via LLM-driven PM3 evidence extraction from scientific literature

**DOI:** 10.1093/bioinformatics/btaf382

**Published:** 2025-06-30

**Authors:** Shumin Li, Yiding Wang, Chi-Man Liu, Yuanhua Huang, Tak-Wah Lam, Ruibang Luo

**Affiliations:** Department of Computer Science, School of Computing and Data Science, University of Hong Kong, Hong Kong, 999077, China; School of Biomedical Sciences, University of Hong Kong, Hong Kong, 999077, China; Hong Kong Genome Institute, Hong Kong, 999077, China; Department of Computer Science, School of Computing and Data Science, University of Hong Kong, Hong Kong, 999077, China; Department of Computer Science, School of Computing and Data Science, University of Hong Kong, Hong Kong, 999077, China; School of Biomedical Sciences, University of Hong Kong, Hong Kong, 999077, China; Department of Statistics and Actuarial Science, The University of Hong Kong, Hong Kong, 999077, China; Center for Translational Stem Cell Biology, Hong Kong Science and Technology Park, Hong Kong, 999077, China; Department of Computer Science, School of Computing and Data Science, University of Hong Kong, Hong Kong, 999077, China; Department of Computer Science, School of Computing and Data Science, University of Hong Kong, Hong Kong, 999077, China; Hong Kong Genome Institute, Hong Kong, 999077, China

## Abstract

**Motivation:**

Rare diseases affect over 300 million people worldwide and are often caused by genetic variants. While variant detection has become cost-effective, interpreting these variants—particularly collecting literature-based evidence like ACMG/AMP PM3—remains complex and time-consuming.

**Results:**

We present AutoPM3, a method that automates PM3 evidence extraction from literatures using open-source large language models (LLMs). AutoPM3 combines a Text2SQL-based variant extractor and a retrieval-augmented generation (RAG) module, enhanced by a variant-specific retriever and fine-tuned LLM, to separately process tables and text. We curated PM3-Bench, a dataset of 1027 variant-publication evidence pairs from ClinGen. On openly accessible pairs, AutoPM3 achieved 86.1% accuracy for variant hits and 72.5% recall for *in trans* variants—outperforming other methods, including those using larger models. We uncovered the effectiveness of AutoPM3’s key modules, especially for variant-specific retriever and Text2SQL, through the sequential ablation study. AutoPM3 located evidence in 76 s, demonstrating that open-source LLMs can offer an efficient, cost-effective solution for rare disease diagnosis.

**Availability and implementation:**

AutoPM3 is implemented and freely available under the MIT license at https://github.com/HKU-BAL/AutoPM3.

## 1 Introduction

Rare diseases remained a formidable public health challenge affecting ∼6% of the global population with ∼8000 known diseases (Taruscio and Gahl 2024). A key factor in diagnosing is the identification of the genetic causes. While the rapid development of whole-genome sequencing (WGS) has made accurate variant detection more affordable, reaching a diagnosis for rare diseases can still be challenging, due to the intrinsic small sample size and limited current understanding from genetic variants to their functional consequences.

**Figure 1.**
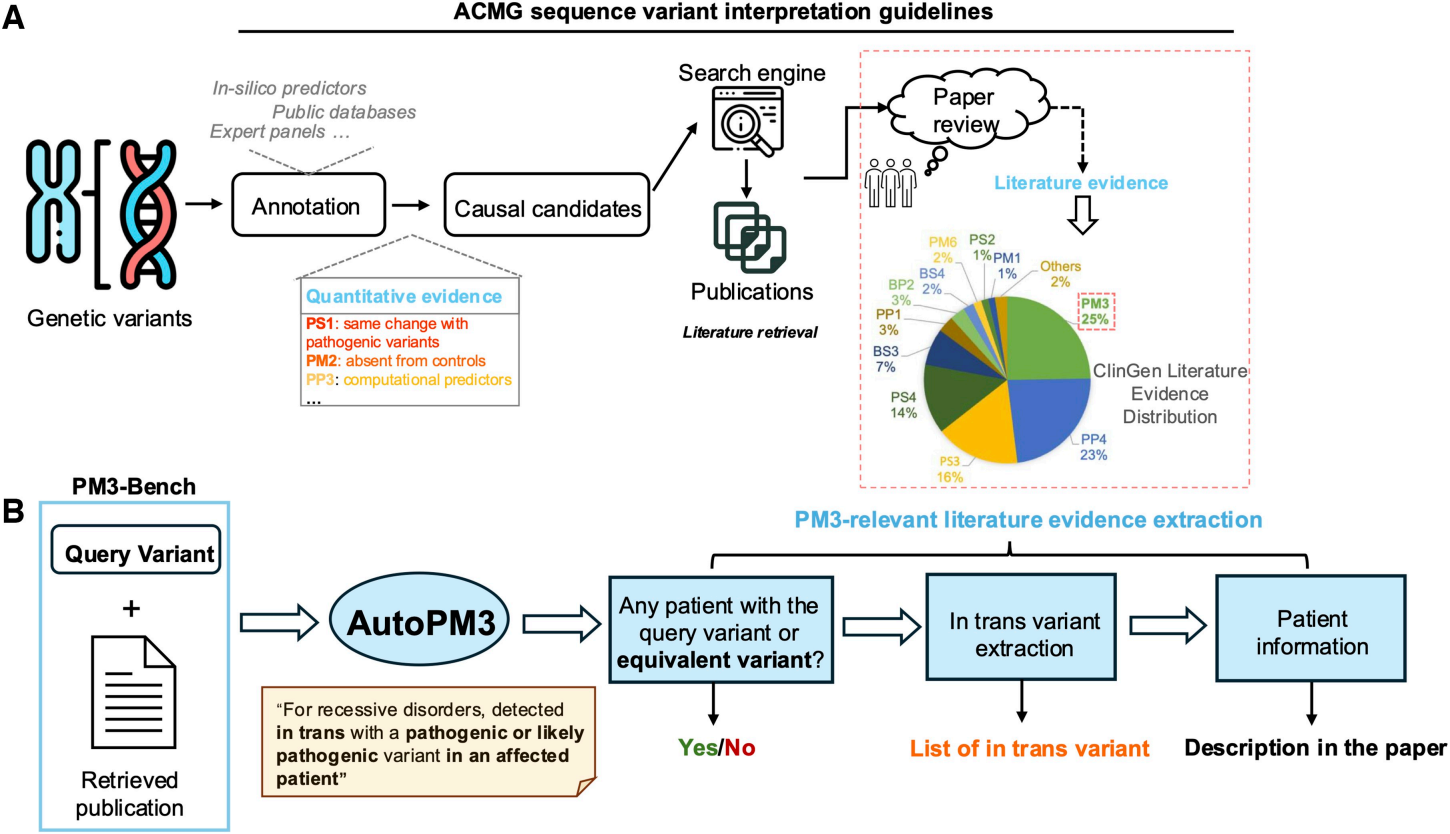
Overview of the variant interpretation workflow and the AutoPM3 system. (A) The commonly adopted manual workflow for variant interpretation, involving variant annotation using external databases and tools, followed by manual literature evidence collection. (B) The concept of the AutoPM3 system, which aims to automate the literature evidence collection step. Given a pair of a publication and a query genetic variant, AutoPM3 can extract relevant information from the literature, including whether the variant was reported, details about in trans variants, and potentially relevant patient information.

The current clinical approach relies on an evidence-based framework, primarily based on the 2015 guidelines published by the American College of Medical Genetics (ACMG) and the Association for Molecular Pathology (AMP) (Richards *et al.* 2015). As illustrated in Fig. 1A, the typical workflow for variant classification involves two major steps: variant annotation and literature review. Variant annotation often involves quantitative indicators, such as allele frequencies in populations (PP3), computational pathogenicity scores [PM2, e.g. REVEL (Ioannidis *et al.* 2016)], etc. These analyses are supported by platforms like Exomiser (Smedley *et al.* 2015), Genomiser (Smedley *et al.* 2016) and Varsome (Kopanos *et al.* 2019), which integrate multiple genomic databases [e.g., GnomAD (Karczewski *et al.* 2020), ClinGen (Rehm *et al.* 2015)] and *in silico* predictors, providing users with an interactive interface for curation. While these tools significantly enhance the efficiency of quantitative variant interpretation, they do not support automated literature mining and are generally limited to basic literature retrieval capabilities.

On the other hand, literature evidence gathers known information from the scientific community. It is widely used in curated variants in ClinGen, with the PM3 criterion being the most frequently utilized, accounting for 25% (Fig. 1A). PM3 is a rule used to support recessive inheritance and applies when the same variant is observed *in trans* with a known pathogenic variant in an affected individual. It refers to the two variants being located on different copies of a gene—one inherited from each parent. In the context of rare disease diagnosis, this typically appears in the literature as case reports or genotype tables showing biallelic genotypes across multiple patients. The Sequence Variant Interpretation (SVI) working group provides a point-based system of PM3, where the evidence strength is determined by the number of probands (i.e. affected individuals), the phasing information and the pathogenicity of the *in trans* variant (Rehm *et al.* 2015). Thus, a necessary step is to collect information on probands reported from the publications that share the same genomic variant and similar clinical syndromes as the target patient. From these, identifying whether a variant is mentioned in the literature is a critical first step. Existing tools such as PubTator (Wei *et al.* 2024) and LitVar (Allot *et al.* 2018, Allot *et al.* 2023) have made important progress by providing variant-centric literature retrieval, serving as a foundational layer for many downstream applications. However, they are primarily focusing on entity recognition and lack the understanding capability for extracting genotype-phenotype relationships or infer pathogenicity. As a result, literature evidence collection for rare disease diagnosis is a time-consuming task that requires expensive expert curation.

Large language models (LLM) have demonstrated remarkable capabilities and become a promising option for understanding and extracting structured knowledge from publications (Dagdelen *et al.* 2024, Huang *et al.* 2024, Polak and Morgan 2024). A recent work demonstrated that GPT4-series models are potentially useful for identifying if one literature contains functional assay data to supports variant classification (Aronson *et al.* 2024). However, the current LLM-based data extractors either use in-context learning techniques or retrieval-augmented generation (RAG) frameworks, which are not optimized for tables, the most information-intensive sections, nor are they designed for ACMG-criteria evidence extraction. Additionally, many of these solutions rely on expensive application programming interface (API) services, their use in local environments is limited.

To bridge this gap, we propose AutoPM3, a method for extracting PM3-relevant evidence from scientific literature, powered by open-sourced LLMs (Fig. 1B). AutoPM3 will determine if the publication mentions the input variant and identify *in trans* variants based on publication of interest. Specifically, the patient information is further aggregated to determine the PM3. AutoPM3 separates the text and tables of the publication and handle them using dedicated LLM-based modules. We employ Text2SQL techniques to sensitively and accurately interpret the table data, while using an optimized RAG system to understand the main texts. There are four key modules in AutoPM3: variant augmentation, Text2SQL-variant-extraction, variant-specific retriever, and model fine-tuning. In addition, we created a benchmarking dataset called PM3-Bench, based on ClinGen comprising 1027 publication-variant data points with ground truth of *in trans* variants and the number of patients.

Through benchmarking experiments, we found that AutoPM3, equipped with lightweight models, significantly outperforms other methods in both variant hit and *in trans* variant detection, with an accuracy of 86.1% for variant hit and a recall rate of 72.5% for *in trans* variants. Through a sequential ablation study, we demonstrated the effectiveness of AutoPM3’s key modules. In conclusion, AutoPM3 is an effective evidence extraction tool for PM3-relevant literature with small models and fast inference time. And this framework is flexible and can be adapted to other ACMG literature-relevant criteria with minimal modifications. To make AutoPM3 accessible and easy to use, we have wrapped it with a user-friendly interface, supporting both online and local usage.

## 2 Materials and methods

### 2.1 PM3-Bench: a curated dataset for PM3 evidence extraction

**Figure 2.**
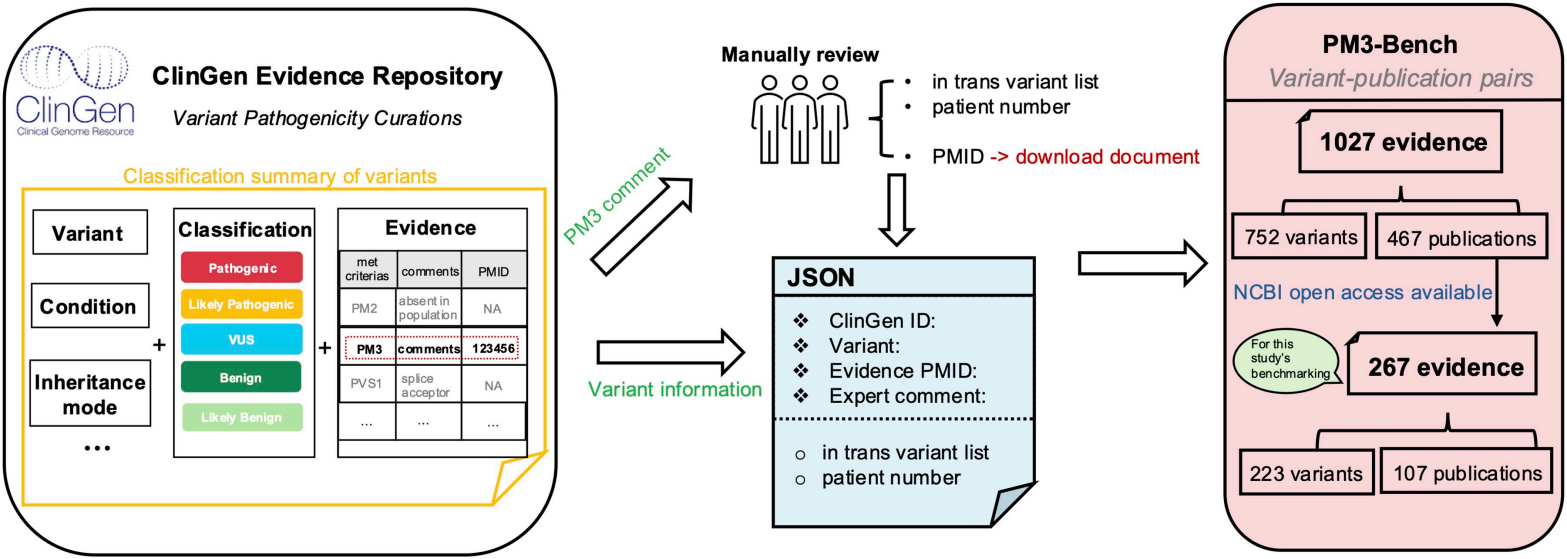
Data curation workflow for the PM3-Bench dataset. The process begins by collecting comments associated with PM3 from the ClinGen database. Comments that could be uniquely traced back to specific publications were retained. These variant-publication pairs were then formatted into a structured data format, and the comments were manually reviewed to extract key information into JSON data. In total, 1027 evidence entries were obtained through this curation process. Of these, 195 entries could be downloaded from the NCBI Open Access subset and were designated as the testing samples for the performance evaluation.

Benchmarking datasets are crucial for developing and evaluating the performance of the computational methods. Although the ClinGen Evidence Repository provides expert-curated assertions, they are written in plain English, posing a difficult challenge for automated evaluation. We created PM3-Bench, a comprehensive dataset for benchmarking PM3 literature evidence extraction (Fig. 2).

First, we collected all variants with applied PM3 criteria from ClinGen. We selected comments associated with PM3 if they were uniquely linked to a single publication, either through a traceable argument or if only one PMID was listed in the comment. For these comments, we manually extracted the *in trans* variants and the number of probands as the ground truth for the corresponding publication.

To evaluate the system’s capability to recognize ambiguous variant notations, we kept the target variants in the format of <transcript identifier>: c.<position reference sequence> <alternate sequence> (e.g., NM_004004.5: c.71G>A). The extracted information was structured into JSON.

The resulting PM3-Bench dataset includes 1027 variant-publication evidence pairs, comprising 752 unique variants and 467 publications. Among these, publications that were available in the PMC Open Access Subset (https://pmc.ncbi.nlm.nih.gov/tools/openftlist/) were downloaded, with 100 publications that were filtered by variants shown in supplementary files only, forming 195 variant-publication evidence pairs. We used these 195 samples as the testing data, while the remaining 605 variant-comment pairs were utilized as fine-tuning samples (Supplementary materials, available as supplementary data at *Bioinformatics* online).

### 2.2 AutoPM3 framework

**Figure 3.**
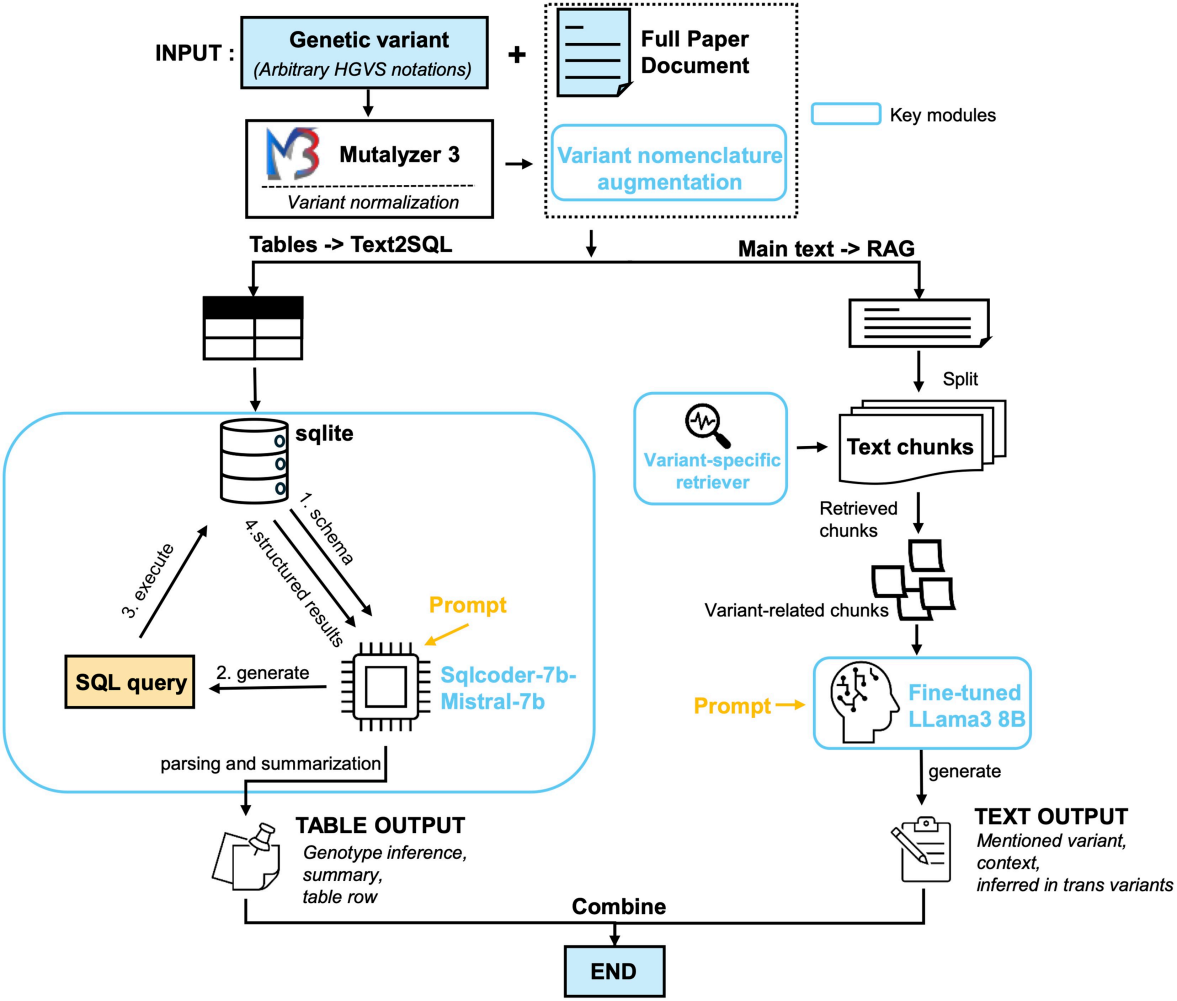
Overview of AutoPM3. The input to the system includes a literature file and a query genetic variant in HGVS format. The variant is first processed by a variant augmentation module, which expands the search space by generating different representations of the same variant. The literature file is then split into tables and main text content. The tables are stored in an SQLite database and processed by the Text2SQL-based variant extractor system, which utilizes a text2SQL core to generate and execute relevant queries and summarize the structured results using Sqlcoder-7B-Mistral-7B. The main text content is processed by an optimized RAG system. The variant retriever component is responsible for fetching variant-related text chunks to be used as context for the backend LLM, the fine-tuned LLaMA 3.8B model on default. The system used predefined queries for variant hit identification and *in trans* variant detection, as well as support customized questions from the user.

AutoPM3 is a document-level PM3 evidence extraction system powered by open-sourced LLMs. The system integrates a Text2SQL and a RAG module to extract PM3-relevant evidence from both table and text contents, separately. As illustrated in Fig. 3, the system accepts a pair of query variant and a publication and the variant augmentation module generates all possible representations of the query variant. These representations are then processed simultaneously by the Text2SQL and the RAG system. The Text2SQL-based variant extractor is designed to extract data from tables through automated database operations and follow-up LLM interpretations. The optimized RAG module efficiently extracts PM3-relevant evidence from texts, which was achieved by our key models of a variant-specific retriever and model fine-tuning.

#### 2.2.1 Variant augmentation

Genetic variants can be represented using either DNA or protein change notations in the HGVS format. To standardize these descriptions, we utilized the Mutalyzer (Lefter *et al.* 2021) to generate possible representations of the variant, such as protein changes. All representations of the query variant were supplied to the downstream RAG and Text2SQL moduels.

#### 2.2.2 Text2SQL

For querying tables, we developed the Text2SQL-based variant-extraction module. Tables were extracted with BioC (Comeau *et al.* 2013), and stored in the SQLite3 database. This module involves three steps: (i) SQL query generation using sqlcoder-7b-Mistral-7B model. (ii) SQL execution and fetching results. (iii) post-processing. The module utilized Text2SQL technique to generates the SQL commands by utilizing table headers and schema, as part of the prompt (see Supplementary Methods, available as supplementary data at *Bioinformatics* online for the full prompt templates and predefined queries). To improve the accuracy, we implemented two measures. First, given the variability in gene representations, the generated SQL is guided by matching variant positions only, followed by subsequent filtering on retrieved records. This filtering is performed by pattern-matching between the fetched string and the variant position. The pattern detects if a previously matched position string appears only among statistical digits, removing false positives like “frequency is 0.93884” in records with query position “388.” For documents containing more than five tables, tables are split into chunks for recursive processing to avoid performance degradation as the LLM’s input context grows. Specifically, for *in trans* variant detection, this module first identifies the rows of the query variant, then extracts its potential ID by the first column. The upstream and downstream rows with the same ID are also extracted for further processing. Finally, we applied the same LLM to the fetched records with specific prompts to generate texts (see Fig. 1, available as supplementary data at *Bioinformatics* online for a representation example for the whole workflow).

#### 2.2.3 Retrieval-augmented generation

RAG is utilized for the querying variant from the main texts of the publications. The text is split into fixed-length chunks containing 1500 characters and each of them has 100 characters overlap, recursiveCharacterTextSplitter of LangChain was used. Given a query variant, we applied a variant-specific retriever to these chunks and retrieved at most five chunks containing the variants as the context. QA chain of LangChain with chain type set as “stuff” was used for answer generations by supplying the retrieved chunks, query variant and the prompts (Supplementary materials, available as supplementary data at *Bioinformatics* online). The backbone LLM was hosted by Ollama (https://ollama.com/), using either our fine-tuned Llama3:8B or other open-sourced LLMs, including Llama3:8B, Llama3:70B, Mistral-Large: 123B. In this framework, two main optimized techniques: variant-specific retriever and fine-tuning, were discussed below:

##### 2.2.3.1 Variant-specific retriever

Similar with variant augmentation, the query variant is first converted to different representations. Examples of representations include DNA change (c.274G>T), protein change (Asp92Tyr), and its shorthand (D92Y). To increase the chance of matching the variant in the text, we create regular expressions from the different representations, e.g. allowing whitespaces, omitting parentheses, interchanging X and the asterisk (*), etc. Successfully matched chunks will be retrieved for downstream query. In case there are too few matched chunks, we will relax the condition to matching digits only (e.g. matching “274” in “c.274G>T”), or even allow some error in the numeric value (e.g. 272–276) since the variant position may not be identical across reference sequences. To increase the chance of matching the variant in the text, we create regular expressions from the different representations, e.g., allowing whitespaces, omitting parentheses, interchanging X and the asterisk (*), etc. Successfully matched chunks will be retrieved for downstream query.

##### 2.2.3.2 Fined-tuned model

Common 8B LLMs tend to generate lengthy responses even with explicit limitation given in the prompt, leads to hallucination and difficulties in evaluation. Thus, succinct answers with clear evidence of PM3 relevant information such as genotype, patient and parental info were preferred. Fine-tuning is feasible to meet this objective with affordable GPU memory size and training efficiency provided by LoRA fine-tuning (Hu *et al.* 2022) on LLama3:8B via Pytorch and Deepspeed training-time acceleration (Rasley *et al.* 2020). With LoRA, parameters from only each transformer layer are modulated by an additional low rank layer. Statistically, low rank matrices only introduce 54 525 952 trainable parameters compared to original LLama3:8B with 8 084 787 200 parameters totally, accounting for 0.674% only. The trainset is extracted from curator reports from ClinGen with relevant comments as inputs and *in trans* variants as true labels, all testing samples were excluded. In this way, the model is trained to further learn representations of gene symbol and variant abbreviation and generate only short and clear answers for target variant and relevant *in trans* variant QA. The example of a training sample is shown in Supplementary materials, available as supplementary data at *Bioinformatics* online. We then fine-tuned the Llama3:8B on three local-rank GPU nodes by torch-distributed-parallel along with Deepspeed at zero3 level. The parameters are as follows: *num_train_epochs* was set at 100, *gradient_accumulation_steps* is set at 8, *learning_rate* is set at 1e-5, *weight_decay* is set at 0.1, *adam beta2* is set at 0.95, *warmup_ratio* is set at 0.01, *lr_scheduler_type* is set at cosine and *model_max_length* is confined to 4096.

### 2.3 Evaluation

We quantitatively evaluate AutoPM3 based on two key aspects. First, we assess variant hit that refers whether the model can correctly identify the presence of the query variant in the publication; hence, it was regarded as the binary classification problem. To evaluate potential hallucinations, we select an equal number of negative variants using real-world variants that were not reported in the publication by random shuffling the query variants (Supplementary materials, available as supplementary data at *Bioinformatics* online). Performance is measured using accuracy and F1 score. Second, we examine whether the model can effectively detect the *in trans* variants based on the query variant. In this case, recall is used as the indicator (Supplementary materials, available as supplementary data at *Bioinformatics* online).

## 3 Results

### 3.1 AutoPM3 effectively detects PM3-relevant evidence

We compared AutoPM3 against the vanilla RAG framework, which employed the same prompts and chunk sizes but lacked AutoPM3’s key modules. Vanilla RAG utilized an ensemble retriever comprising BM25 and PubMedBERT embedding models (https://huggingface.co/NeuML/pubmedbert-base-embeddings) to ensure competitive retrieval performance (Xiong et al. 2024). In this experiment, AutoPM3 utilized two lightweight models: a fine-tuned Llama3:8B for RAG module and a 7B sqlcoder-mistral model for Text2SQL. We compared the performance of vanilla RAG equipped with three open-source LLMs: Llama3:8B, Llama3:70B, and Mistral-large: 123B. We also included PaperQA, a general scientific paper question-answering tool, with Llama3:70B as the backend, for comparison.

**Figure 4.**
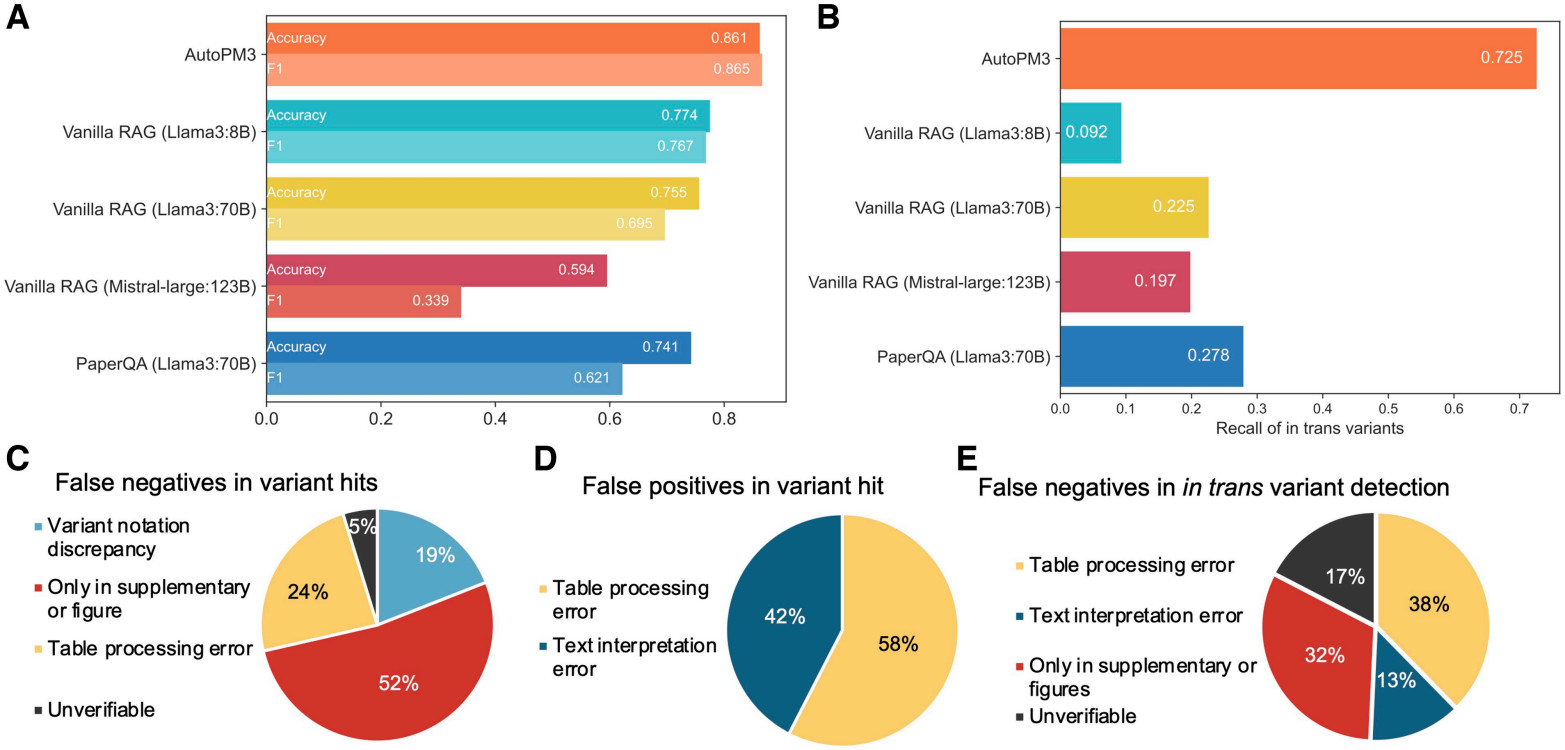
Benchmarking performance of AutoPM3 against baseline methods. (A) Comparison of variant hit performance across AutoPM3, vanilla RAG, and PaperQA. (B) Recall for *in trans* variant identification using the different methods. The input is the pair of positive query variant and literature, and the model generates the responses based on the predefined in trans specific query. (C) Distribution of false negatives in variant hit of AutoPM3. (D) Distribution of false positives in variant of AutoPM3. (E) Distribution of false negatives in *in trans* variant detection of AutoPM3.

We first evaluate the performance of variant hit. Shown in Fig. 4A, AutoPM3 achieved the highest accuracy and F1 score of 0.861 and 0.865, significantly outperforms the second-best method, vanilla RAG (Llama3:8B), with an accuracy of 0.774 and F1 of 0.767. Notably, vanilla RAG and PaperQA using larger models (Llama3:70B and Mistral-Large: 123B) exhibited variant hit accuracy of 10–26% lower than AutoPM3 equipped with small models (8B and 7B), despite being more than nine times larger. However, these larger models achieved the highest specificity, all exceeding 0.96, partly by sacrifcing sensitivity (Table 1, available as supplementary data at *Bioinformatics* online). This discrepancy may result from the vanilla RAG retriever’s insensitivity to genetic variants, leading to more irrelevant text chunks retrieved and causing high specificity and low sensitivity for larger models.

In addition to variant hit accuracy, we evaluated AutoPM3’s effectiveness in identifying the *in trans* variants. We obtained the recall of *in trans* variant detection as the performance metric (Supplementary materials, available as supplementary data at *Bioinformatics* online). Demonstrated in Fig. 4B, AutoPM3 identified 72.5% of *in trans* variants, while all other methods identified less than 30%, highlighting AutoPM3’s effectiveness. Of note, considering cumbersome detection of false hits, recall is the primary metric here for assessing *in trans* variants.

We observed that vanilla RAG (Llama3:8B), whose size can be considered the same as AutoPM3, identified only 9% of the ground truth *in trans* variants, failing in most cases. Even methods using large models like Llama3:70B and Mistral-large: 123B, identified less than 30% of the ground truth *in trans* variants, which was substantially lower than AutoPM3’s recall, that was equipped with only 8B models. These results indicate that for complex inference tasks, large models may not exhibit superior performance compared to small models if the agent system is not optimized, despite that large models usually outperform small models in simpler tasks.

Furthermore, we analyzed the false cases in both variant hit and *in trans* variant detection (Fig. 4C–E). For false negatives in both tasks, we found that a major portion resulted from information appearing only in supplementary materials or figures, which AutoPM3 currently cannot process due to their complex formats and lack of visual interpretation capabilities. A second major source of false negatives was table processing errors, primarily caused by limitations in the table post-processing module. Lastly, we observed a smaller set of text interpretation errors, which we believe could be addressed by integrating more advanced language models with improved reasoning capability.

### 3.2 Ablation analyses uncover key contributors of AutoPM3

Having demonstrated AutoPM3’s superiority over vanilla RAG and PaperQA, we further investigated the contribution of each key module: Text2SQL, variant augmentation, variant retriever, and model fine-tuning. Moreover, we explored how large-scale models like Llama3:70B and Mistral-large: 123B could enhance the performance of the RAG module.

**Figure 5.**
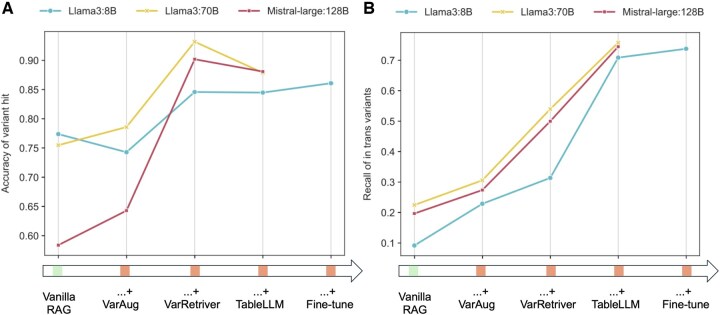
Contributions of AutoPM3 modules on PM3-relevant evidence extraction across different scaled language models. (A) Variant hit identification accuracy when sequentially adding key modules of AutoPM3 to a baseline RAG system using LLaMA 3.8B, LLaMA 70B, and Mistral-Large 123B models. (B) Recall of in trans variant detection when sequentially incorporating the AutoPM3 modules into the RAG systems with the different language model sizes.

We conducted experiments by sequentially adding these modules to three open-sourced models, starting with vanilla RAG (Tables 2 and 3, available as supplementary data at *Bioinformatics* online). For the variant hit, shown in Fig. 5A, variant augmentation resulted in minor accuracy increases for Llama3:70B and Mistral-Large: 123B but decreased accuracy for Llama3:8B. This is because variant augmentation expands the search space, enhancing sensitivity but also increasing hallucination risks. While larger models maintained stable specificity, Llama3:8B’s specificity decreased from 0.8 to 0.692, with a 3% sensitivity increase. Furthermore, significant performance improvements were observed when adding the variant retriever to the Llama3:70B and Mistral-large: 123B models: the sensitivity of Llama 3:70B and Mistral-Large: 123B increased from 0.786 to 0.932 and 0.643 to 0.902, respectively. And Llama 3:8B’s specificity increased from 0.692 to 0.81. These results suggest the variant retriever module is crucial for 70B and 123B models to produce accurate answers and reduce hallucinations in 8B models. Next, we integrated the Text2SQL with the RAG system. The texts were processed by the RAG system with variant augmentation and variant retriever modules, while the table components were handled by Text2SQL. The performance of Llama 3:8B remained stable, while a slight fluctuation in accuracy was observed for Llama 3:70B and Mistral-large: 123B. This suggests that for a simple binary task, large models already possess a certain level of table interpretation capability, reducing the additional benefit provided by Text2SQL. Finally, we fine-tuned the Llama 3:8B model for evidence extraction, further increasing the accuracy of the 8B model-based system to 0.861.

For the *in trans* variant detection task, we observed a consistent performance increase when adding the variant augmentation and variant retriever modules for all models (Fig. 5B). The variant retriever contributed the most significant performance jump, increasing recall by approximately 20% for 70B and 123B models and 10% for the 8B model. This highlights the importance of accurate text chunks retriever. Notably, equipped with these two modules, both Llama 3:70B and Mistral-large: 123B achieved over 50% recall, while Llama 3:8B remained around 31%, indicating a positive relationship between model size and *in trans* variant detection. We further integrated Text2SQL into the system, consistently increasing the recall by approximately 20% for Llama 3:70B and Mistral-large: 123B. The results indicated that although these two models demonstrated a certain level of logical inference for table content, their performance was inferior to the dedicated Text2SQL module, necessitating its inclusion into the system. This effect was amplified for Llama 3:8B, whose table content comprehension capability was even weaker, resulting in a substantial recall increase by using Text2SQL, from 0.314 to 0.709. In the final step, we fine-tuned the Llama 3:8B model to improve its understanding of genetics publications, boosting the overall *in trans* variant detection recall to approximately 0.725.

In addition, we examined the relative contributions of tables and texts to the outputs of AutoPM3. Based on a comparison of results from the Text2SQL and RAG modules (see Fig. 2, available as supplementary data at *Bioinformatics* online), we found that tables contributed more true positives than text for both variant hit and *in trans* variant detection tasks. The effect was especially significant for *in trans* variant detection, where patient genotype information was typically embedded in tables. This indicates the importance of incorporating table-specific reasoning modules for accurate clinical evidence extraction.

### 3.3 Case study of AutoPM3

To allow AutoPM3 to be exploited more easily, we have integrated AutoPM3 with an intuitive browser-based interface known as Streamlit. This interface can be accessed online at https://www.bio8.cs.hku.hk/autopm3-demo/ or deployed locally. The public demo version utilizes AutoPM3 equipped with the RAG modules of fine-tuned Llama3:8B, while users have the option to switch to larger models in their local deployment.

**Figure 6.**
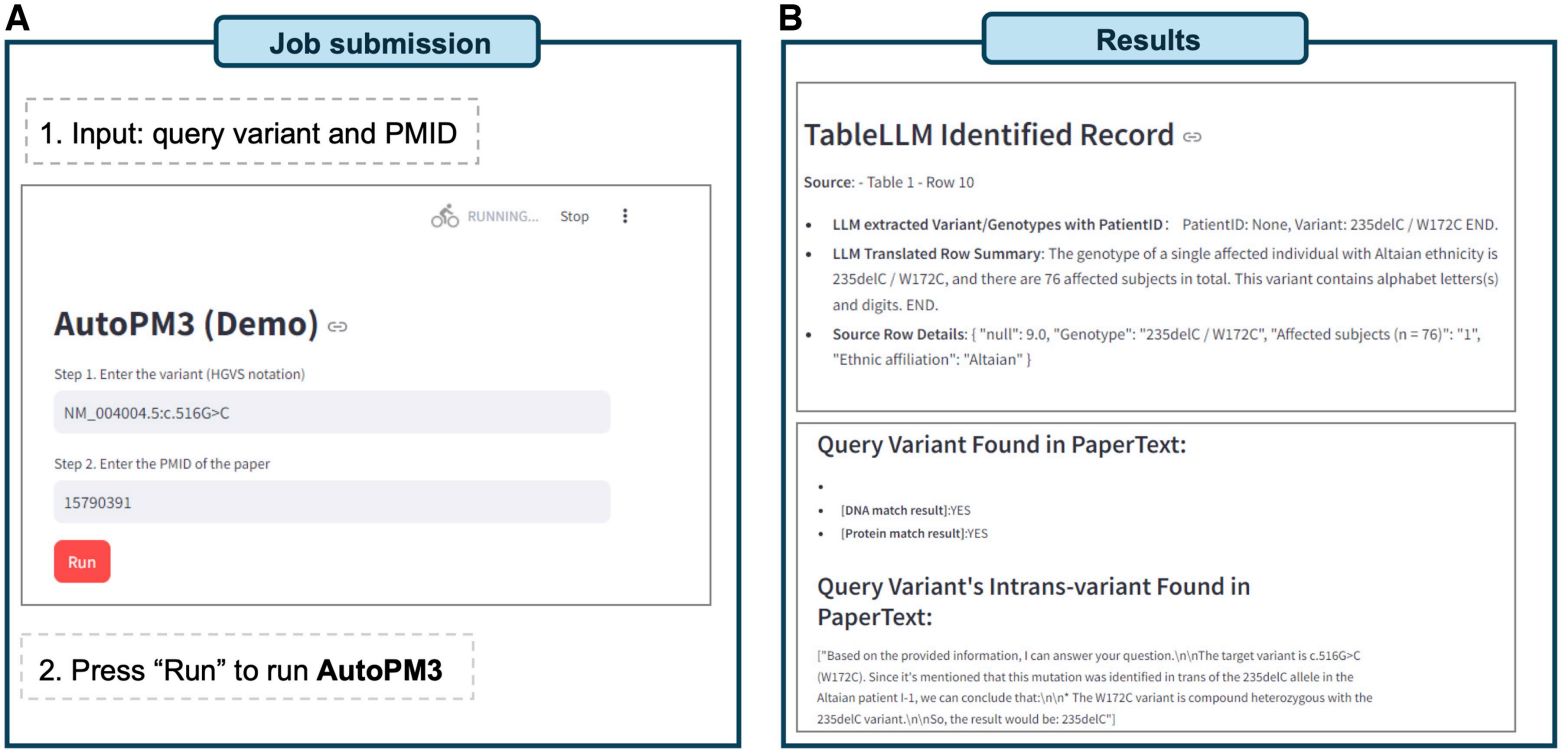
Overview of the AutoPM3 interface and use cases. (A) Illustration of the input and usage of AutoPM3. (B) Example outputs generated by AutoPM3, showing results from both Text2SQL and the RAG module.

As illustrated in Fig. 6A, the system requires two inputs: the query variant in HGVS notation and the PMID of the publication. The system retrieves the corresponding publication using the NCBI Open Access API and then executes AutoPM3 in the backend (Fig. 6B).

The results are divided into two sections based on Text2SQL and RAG modules. For Text2SQL, each queried result is presented in four lines of information: (i) The source index of the results, e.g. as shown in Fig. 6B, demonstrates “Source: Table 2, row 3”; (ii) inferred genotypes per patient; (iii) summarized information of identified rows; (iv) the original row in the table. It ensures that PM3-relevant information can be easily traced back to the original tables. Similarly, for the RAG module, which processes text, the rationale for the generated content follows the same structure. The results are presented based on two predefined queries: variant hit and *in trans* variant detection. And the original context of the query variant is extracted by the LLM for comprehensive consideration.

To illustrate AutoPM3’s utility in real-world curation, we conducted a case study using a newly released ClinGen variant (NM_000059.4: c.7796A>G) not included in the PM3-Bench. Based on the relevant publications retrieved using LitVar 2.0 (search term: “NM_000059.4: c.7796A>G”) (Allot *et al.* 2023), AutoPM3 correctly identified the PM3-relevant article and accurately detected the *in trans* variant (c.1813dup), consistent with the ground truth (Table 4, available as supplementary data at *Bioinformatics* online). While one of the nine retrieved publications produced a false positive from a table, we note that such cases can be easily identified and filtered out in practice by reviewing the structured summaries provided by AutoPM3. Generally, this demonstrates the system’s potential to support prospective variant interpretation.

Overall, using a GPU Nvidia RTX 4090 for our benchmarking pairs, AutoPM3 locates PM-relevant evidence in an average of 74 s per variant-publication. This significantly reduces the time required for professionals to read and extract information from a paper, which typically takes over an hour.

## 4 Discussion

In this study, we present AutoPM3, a framework that utilizes LLMs for the PM3-relevant evidence extraction from the literature. Additionally, we compiled PM3-Bench, a dataset comprising variant-publication pairs with curated ground truth for this task. Through the integration of AutoPM3 and PM3-Bench, we explore the potential and effectiveness of an LLM-based approach to enhance the ACMG-guided variant interpretation workflow in literature evidence collection.

The core of AutoPM3 involves integrating two modules for independently processing tables and text. We framed the table-based evidence extraction as a database query problem and developed the Text2SQL-based variant-extraction. This component generates SQL executable commands using the sqlcoder-Mistral-7B model, enabling database operations. The results retrieved from the tables are translated into summarization texts using the same LLM. Concurrently, we employed the RAG to extract evidence from the main text. We optimized the RAG by developing a variant retriever and fine-tuning the backend LLM, Llama3:8B. The combination of Text2SQL and RAG within AutoPM3 demonstrates effective document-level evidence extraction, significantly outperforming vanilla RAG and PaperQA, even when these systems were powered by models nine times larger. Powered by two lightweight models, AutoPM3 achieves fast response times and lower hardware requirements for local deployment.

Furthermore, we conducted a sequential ablation analysis of AutoPM3’s key modules. Our analysis revealed that the variant retriever integrated within the RAG is crucial for enhancing performance in variant hit and *in trans* variant detection. The variant retriever effectively locates text chunks containing the query variant, unlocking the potential of larger models (70B and 123B) and achieving approximately a 20% increase in performance. Text2SQL also plays a vital role, particularly in *in trans* variant detection. Although larger models perform better in table understanding, their recall of *in trans* variants is still capped at around 0.5. In contrast, our Text2SQL reformulates the task as a database query problem, effectively addressing this limitation and improving recall by over 20% when integrated with RAG across all model sizes. While the RAG system of AutoPM3 can be further improved by using larger models as the backend, we did not include the 70B and 123B models in our default settings due to their increased running time and hardware requirements. However, the inherent support for these models is maintained in the AutoPM3.

In addition to the quantitative indicators for variant hits and *in trans* variants, AutoPM3 also summarizes patient information related to the query variant. Our principle is to extract PM3-relevant evidence rather than directly classifying whether a paper meets PM3 criteria. This approach allows users to review the results and source texts to make the final decision.

One limitation we observed is the file format of the publications. Although NCBI XML files were used in our experiments, which provide structured access to article content (including tables), the common format of scientific literature is PDF. Even if AutoPM3 is applicable to PDFs, its performance partly depends on the accuracy of the PDF parser for extracting tables. In our evaluation of several popular free PDF parsers, we observed that table extraction was often unreliable, with issues such as missing headers, merged cells, or truncated rows, all of which impair Text2SQL processing. However, with the development of more accurate parsers or broader adoption of structured formats like XML in scientific publishing, this issue can be addressed in the future. A second limitation is the lack of support for supplementary materials and figures of the literatures, which often contain critical evidence. Processing these sources correctly requires substantial engineering effort to handle a variety of formats, as well as methodological advances in visual interpretation. This suggests that a multimodal, LLM-based system is necessary for fully capturing evidence from all parts of a publication. Finally, we aim to explore linking external pathogenicity databases to AutoPM3, which can justify the pathogenicity of identified *in trans* variants.

With lightweight models, AutoPM3 can be easily installed and run locally within a clinical center, with minimum costs. For instance, using an Nvidia RTX 4090, it averages 74 s per variant-publication. We expect these advancements to facilitate the literature evidence collection for the variant interpretation, thus supporting rare disease diagnosis.

## Supplementary Material

btaf382_Supplementary_Data

## Data Availability

Source code of AutoPM3 and PM3-Bench are accessible at: https://github.com/HKU-BAL/AutoPM3 and has been archived at Zenodo (https://zenodo.org/records/15629004). The GGUF format of fine-tuned LLaMA3 model is available at: http://bio8.cs.hku.hk/AutoPM3. The online web server is accessible at: https://www.bio8.cs.hku.hk/autopm3-demo/.
