# Supplementary material for: AutoPM3: enhancing variant interpretation via LLM-driven PM3 evidence extraction from scientific literature
Source: Bioinformatics. 2025 Jun 30;41(7):btaf382. doi: 10.1093/bioinformatics/btaf382 (PMC12263107; doi:10.1093/bioinformatics/btaf382)
Supplement: btaf382_Supplementary_Data [file btaf382_supplementary_data.zip › Supplemenary Materials.pdf]

Supplementary Material

AutoPM3: Enhancing Variant Interpretation via LLM-driven PM3 Evidence Extraction from Scientific Literature

Supplementary Figures

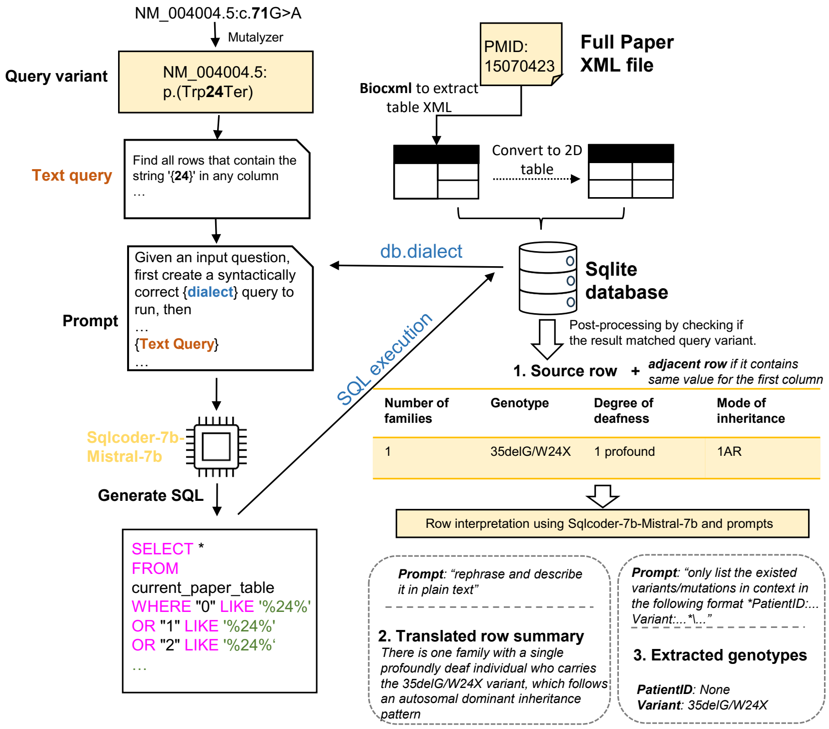

Supplementary Figure 1. Example of Text2SQL-based variant extraction from literatures. The complete prompt templates and predefined queries are provided in the Supplementary Methods.

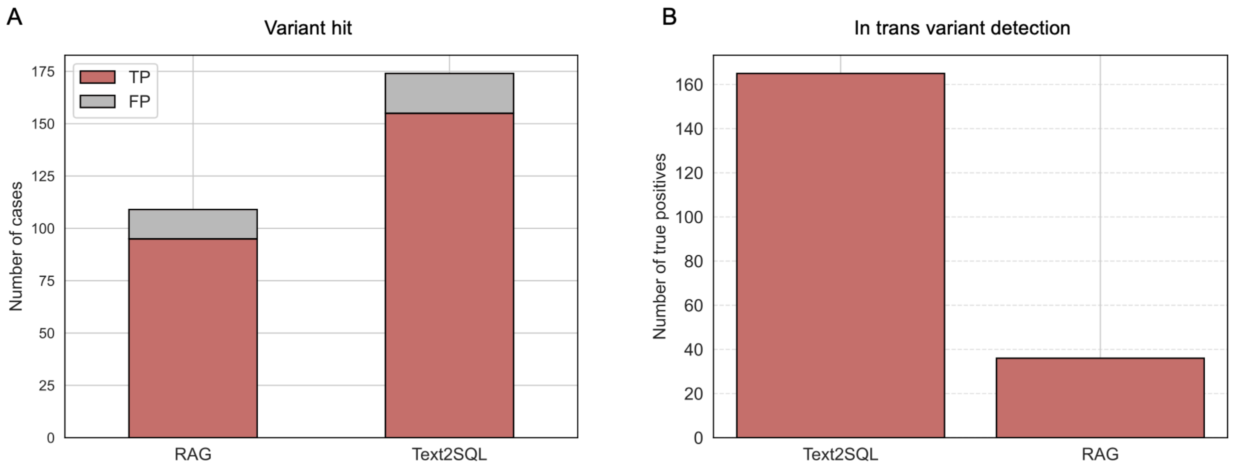

Supplementary Figure 2. Contribution of Text2SQL and RAG modules to variant hit and in trans variant detection. A, Number of true positives (TP) and false positives (FP) identified by the RAG and Text2SQL modules for the variant hit. B, Number of true positives identified by each module for the in trans variant detection.

Supplementary Methods

Prompts and predefined queries of RAG model

The prompt of RAG module for both variant hit and *in trans* variant detection:

```
<|begin_of_text|><|start_header_id|>system<|end_header_id|>
You are a specialist in biogenetics, answer only based on user's input!<|eot_id|>
<|start_header_id|>user<|end_header_id|>
The variant in HGVS format is {question}, don't include this in your answer if condisering
compound het variants.
Given the context: '{context}' and target variant {c_variant}. Answer the question:
{proposedQuestion}<|eot_id|>.
<|start_header_id|>assistant<|end_header_id|>
```

The predefined query for the task of variant hit:

```
Does the paper mention the queried variant ({current_variant}) and what is the
surrounding context? if such variant is existed, say *YES* at first otherwise say *None*
(focus on variant: {current_variant})
```

The predefined query for the task of *in trans* variant detection:

```
If {current_variant} is compound heterozygous with another variant, name it; if
{current_variant} is homozygous, say homozygous; if no related variant is found, say
*None*. List all results separated by comma.
```

Prompts and predefined queries of Text2SQL

The prompt of Text2SQL for generating the SQL commands:

```
Given an input question, first create a syntactically correct {dialect} query to run, then
look at the results \
of the query and return the answer. Strict your query to a short one and dont give a long
answer. *Never* use limitation to limit your query like: LIMIT {top_k} except user asked for
certain row.\nWhen no specific column names are given, you can check for the answer in
all columns using "OR" operator.\n\n\
Unless exactly match is required by user, use LIKE other than = in the query\n\
Never sort the results. If user asks for certain row, use LIMIT operator!\n\
Never give a sql that will return all content in the table if not explicitly asked\n\
Only give one query ended with \';\' eachtime!\n\
```

```
Carefully check the statement after WHERE clause, don\'t mix up column_name with user\'s query string, and keep the string integral for matching!\n\
When using LIKE operator, note to put column names on the left and query string on the right, don\'t reverse it\n\
Don\'t forget to append ; at the end of query and no order is needed!\n\nPay attention to use only the column \
names that you can see in the schema description. Be careful to not query for columns that do not exist. Also, pay \
attention to which column is in which table.\n\nUse the following format:\n\nQuestion: Question here\nSQLQuery: SQL \
Query to run\nSQLResult: Result of the SQLQuery\nAnswer: Final answer here\n\nOnly use the \
following tables:\n{table_info}\n\nQuestion: {input}

get the first row in table {current_table}? (take the result given by SQLResult:)"

search for the string: '{query_variant}' through every column in table {current_table} using OR? (find all, no limit, column names should be like 0,1,2 as u can see in the schema)"
```

The predefined queries for generating the SQL commands:

```
search for the string: '{query_variant}' through every column in table {current_table} using OR? (find all, no limit, column names should be like 0,1,2 as u can see in the schema)
```

```
find all rows that contain the string '{query_variant}' in any column (don\'t only consider one column) (check all columns in table {current_table}) (find all, no limit)"
```

```
find all the rows that contain {query_variant} (query all columns in table {current_table} using OR) (find all, no limit)
```

The prompt of Text2SQL for convert the database query results into texts:

```
### System:
You are reading the structured data given in the Context and try to rephrase it in plain
text. In each line, the attribute name(header) is on the left of *:*, then corresponding
attribute value is on the right.

### Context:
{tableData}

### User:
Each variant/mutation must contain alphabet letters with several digits, don't make up
non-existed variants/mutations.
Limit your answer under 25 words.
Stop the answer by the word *END*.
Please read the above provided structured data in context and just answer the given
question in short plain text. Question: {question}'\
### Response:
```

Predefined query for summarization of the fetched results:

```
rephrase and describe it in plain text
```

Predefined query for extracting variant and patient information from the fetched results:

```
only list the existed variants/mutations in context in the following format *PatientID:...
Variant:...*\nif no patient is explicitly mentioned put *PatientID:None* and don't mix up
with variants/mutations. If no variants/mutations is explicitly mentioned put
*Variant:None*
```

Fine-tuning data sample


```
{
  "conversations": [
   {
    "from": "user",
    "value": "Given the context: 'Two missense mutations causing mild HPA associated with haplotype 12 88 swedish families. 93 children picked up by NBS A322G / R408Q – 300uM/L A322G / R408W – 250uM/L A322G / R408W – 360uM/L A322G / R252W – 250uM/L' and target variant in two format c.965C>G/p.(Ala322Gly). If target variant is compound heterozygous with another variant, name it; if the target variant is homozygous, say homozygous; if no related variant is found, say NA. List all results seperated by comma."
   },
   {
    "from": "assistant",
    "value": "The variants contains:R408Q,R408W,R252W."
   }
  ],
  "id": 1
 },
```

Evaluation

Variant hit

We evaluated the variant hit task as a binary classification problem, where the goal was to determine whether a publication mentioned the queried genetic variant. The positive samples were obtained from the PM3-Bench dataset, which were collected from ClinGen. To generate the negative samples, we randomly selected variants mentioned in a different publication and located in a different gene from the true variant set. We then performed regular expression matching to ensure that these negative variants were not mentioned in the corresponding publication. The final testing dataset consisted of 195 positive pairs and 195 negative pairs.

For the evaluation, we used the following approaches: for Text2SQL, if Text2SQL reported any queried results, we considered it as a positive answer; otherwise, it was treated as a negative answer. For RAG, the system generated responses based on the question "*Does the context mention the queried variant {variant name} and what is the surrounding context? if such variant exists, say *YES* at first otherwise say *None* (focus on variant: {variant name})*". If the response contained the word "Yes", we considered it as a positive answer; otherwise, it was treated as a negative answer. If either Text2SQL or RAG module reports a positive response, we consider it as the positive answer. The accuracy, sensitivity and specificity, precision and F1 of variant hit were calculated as below (note, the numbers of positive and negative samples are equal):

$$\text{Sensitivity} = \frac{\text{No. of correct pair(publication, positive variant)}}{\text{No. of pair(Publication, positive variant)}} \tag{1}$$

$$\text{Specificity} = \frac{\text{No. of correct pair(publication, negative variant)}}{\text{No. of pair(Publication, negative variant)}} \tag{2}$$

$$\text{Accuracy} = \text{Sensitivity} \times 0.5 + \text{Specificity} \times 0.5 \tag{3}$$

$$\text{Precision} = \frac{\text{Sensitivity}}{\text{Sensitivity} + (1 - \text{Specificity})} \tag{4}$$

$$\text{F1} = 2 \times \frac{\text{Sensitivity} \times \text{Precision}}{\text{Sensitivity} + \text{Precision}} \tag{5}$$

In trans variant detection

In trans variants are a key factor of PM3-relevant evidence. For our testing set, which contains 195 variant-publication pairs, with including total ground truth 294 *in trans* variants.

For evaluation, we have adopted a semi-automated way for evaluating the performance of *in trans* variant detection as the following procedure:

Given:

- Ground truth *in trans* variants GT
- Responses of the system R

1. For each ground truth *in trans* variant $g \in GT$:
   1.1. Generate all possible formats F for g, including DNA change, protein change, and homozygous state if applicable.
   1.2. Initialize flags $good\_it$ and $suspect\_hit$ to empty lists.
   1.3. For each response $r \in R$:
      1.3.1. If any format $f \in F$ is found in r, append *f* in $good\_hit$.
      1.3.2. If $good\_hit$ is False and the position of any format $f \in F$ matches the position in r within a tolerance, append f in $suspect\_hit$.
   1.4. Save the $good\_hit$ and $suspect\_hit$.

2. Manually review the responses with at least one $good\_hit$ or $suspect\_hit$, and update the count of correctly identified *in trans* variants accordingly.

The recall was calculated based on the manually reviewed results as:

$$\text{Recall} = \frac{\text{No. of correctly identified } in\ trans \text{ variants}}{\text{No. of ground truth } in\ trans \text{ variants}} \tag{1}$$

Noted that in our benchmarking samples, there are 294 ground truth *in trans* variants.

Supplementary Table 1. Variant hit performance of AutoPM3, vanillaRAG, and PaperQA.

**Methods**	**Sensitivity**	**Specificity**	**Precision**
AutoPM3	0.892	0.83	0.839
VanillaRAG (Llama3:8B)	0.748	0.8	0.789
VanillaRAG (Llama3:70B)	0.558	0.953	0.922
PaperQA (Llama3:70B)	0.468	0.962	0.924

Supplementary Table 2. Variant hit performance of different models by sequentially add key AutoPM3's key modules

Model	Sequentially added modules	Sensitivity	Specificity	Accuracy
Llama3:8B	Vanilla RAG	0.748	0.8	0.774
	Variant augmentation	0.794	0.692	0.743
	Variant retriever	0.882	0.81	0.846
	Text2SQL	0.815	0.876	0.845
	Fine-tuning	0.892	0.83	0.861
Llama3: 70B	Vanilla RAG	0.558	0.953	0.755
	Variant augmentation	0.62	0.953	0.786
	Variant retriever	0.876	0.989	0.983
	Text2SQL	0.887	0.871	0.879
Mistral-Large: 128B	Vanilla RAG	0.205	0.984	0.594
	Variant augmentation	0.292	0.994	0.643
	Variant retriever	0.81	0.994	0.902
	Text2SQL	0.887	0.876	0.881

Supplementary Table 3. *In trans* variant detection performance of different models by sequentially add key AutoPM3's key modules.

**Model**	**Sequentially added modules**	**Recall**
Llama3:8B	Vanilla RAG	0.092
	Variant augmentation	0.229
	Variant retriever	0.314
	Text2SQL	0.709
	Fine-tuning	0.725
Llama3: 70B	Vanilla RAG	0.225
	Variant augmentation	0.306
	Variant retriever	0.540
	Text2SQL	0.758
Mistral-Large: 128B	Vanilla RAG	0.197
	Variant augmentation	0.274
	Variant retriever	0.5
	Text2SQL	0.745

Supplementary Table 4. AutoPM3 Results for the variant of NM_000059.4:c.7796A>G.

PMID	Variant hit	In trans variant	Used in ClinGen
34504103	Yes	c.1813dup	Yes
39779857	Yes	No	No
33428613	Yes	False table hit	No
26913838	No	No	No
31911673	No	No	No
29884841	No	No	No
32377563	No	No	No
28993434	No	No	No
36922933	No	No	No
